# Innovation, Adaptation, and Human Dignity in Assistive Robotics in Amyotrophic Lateral Sclerosis: A Rehabilitation Medicine Perspective

**DOI:** 10.26502/jbb.2642-91280207

**Published:** 2026-02-05

**Authors:** Rafaelle B Azarraga, Mark C Jackson, Marcel P Fraix, Devendra K Agrawal

**Affiliations:** 1 Departments of Translational Research, Western University of Health Sciences, Pomona, California 91766 USA; 2 Physical Medicine and Rehabilitation, Western University of Health Sciences, Pomona, California 91766 USA

**Keywords:** Adaptation, Amyotrophic Lateral Sclerosis, Assistive Robotics, Augmentative and Alternative Communication, Cognitive Interfaces, Emerging Health Technologies, Functional Independence, Human-Machine Integration, Mobility Support, Quality of Life in ALS

## Abstract

Amyotrophic Lateral Sclerosis (ALS) is a rapidly progressive motor neuron disease that heavily impacts a person’s ability to perform activities of daily living, affecting mobility, function, and communication drastically. These complications present a daunting obstacle for familial support systems and physicians to manage. Although survival prognosis in ALS patients has moderately improved with the advent of ALS specialty clinics, this illness persists as a grim diagnosis with no current cure or hope for recovery, simply an inescapable decline. However, assistive robotics provides a promising opportunity in empowering patients to retain control of their lives. This comprehensive review explores the current developments in assistive robotics for ALS patients across various aspects of daily living. Our review accentuates how robotics can make a significant impact on quality of life, preserving physical capabilities and patient agency. Potential barriers have also been identified, such as cost, accessibility and ethical considerations. However, there is limited information with case-controlled studies and robust research addressing ALS from this scope. Within the lens of rehabilitation, these technologies present the opportunity to preserve *autonomy*, the ability to still care for oneself, to perform the “everyday” tasks, the things that make life still meaningful. This critical review highlights the humanistic potential that the future of this emerging field holds; to innovate and bridge the technical with the personal; and to approach decline with empathy, adaptability and respect.

## Introduction

Amyotrophic Lateral Sclerosis (ALS) is a relentless neurodegenerative disease that gradually robs individuals of movement, speech, and independence, while leaving cognition largely intact. For patients and families, it is both medical and deeply human, a daily negotiation between adaptation and loss. Despite advances in multidisciplinary ALS clinics and improved symptom management, the condition remains incurable and profoundly life-altering [1]. Within this landscape, assistive robotics offers not a cure but a means to sustain dignity and agency. New innovations in assistive technology such as robotic arms, wearable exoskeletons, eye-tracking systems, and brain-computer interfaces extend the body’s ability to engage with the world. Studies show that robotic assistance can improve independence, ease caregiver burden, and strengthen the sense of control of a patient [2–4]. Devices that support mobility help compensate for weakness and fatigue [5–9], while communication systems restore expression and social connection even in advanced disease [10–16]. Recent progress in neuroprosthetic voice synthesis and rapid speech decoding suggests that human communication can persist even as the body declines [17–24]. In this light, robotics becomes more than mechanical design; it becomes an act of empathy in motion. This review explores how assistive robotics supports mobility, communication, and quality of life in ALS, proposing that innovation guided by compassion can redefine what it means to rehabilitate within decline.

### Pathophysiology and Clinical Context

Amyotrophic Lateral Sclerosis (ALS) is a relentlessly debilitating disease that impacts movement and independence. Clinically, this presents as rapid onset loss of voluntary motor control, demonstrating both upper and lower motor signs. Over time, patients lose muscle strength to the point of paralysis as they gradually lose the ability to walk, speak, swallow and ultimately breathe of their own volition. Notably, consciousness and intellect remain intact, creating a grim diagnosis as a patient is consciously aware of their steep decline. ALS remains a disease of mixed etiology [1–2]. Most cases occur without clear genetic cause, though recent studies have linked one in ten cases to mutations in genes such as SOD1, *TARDBP*, *C9orf72*, and *FUS.* The underlying physiology involves a complex cascade of neuronal destruction associated with oxidative stress, inflammation and glutamate toxicity [3]. Though there is no cure, the current standard of treatment utilizes riluzole and edaravone to slightly slow disease progression [4]. Median survival from the time of diagnosis is estimated to be three to five years. Additionally, the advent of ALS specialty clinics has extended both the quality of life and longevity for many patients [6]. However, the survival statistics do not encompass the entirety of the picture. The core of the disease and how it impacts a person’s livelihood lies in its steady erosion of autonomy both for their patients and their caregivers.

From a holistic perspective, the clinical goal for ALS patients shifts to preservation; to maintaining a person’s ability to control their body as much as possible. Due to the current innovations in robotics and artificial intelligence (AI) technology, assistive devices have grown more advanced. This has paved the way for an emerging opportunity in providing care for patients who have lost motor control. Already, assistive robotics have made incredible bounds in the field of stroke recovery and rehabilitation [19–20]. Much less studied, is the impact these technologies can have on ALS patients.

## Methods

Utilizing various research databases such as PubMed, IEEE Xplore, Scopus, Google Scholar, SciSpace and arXiv, this review analyzed 119 peer-reviewed articles. Our inclusion criteria involved peer-reviewed studies published between 2010–2025 in English language, with ALS-specific applications, prioritizing primary research studies over review articles. Meanwhile exclusion criteria consist of ruling out purely engineering simulation papers without user data and non-ALS populations unless technology is directly transferable.

Though the distribution included a sparse amount of review articles and meta-analyses, the focus of this review encompassed pilot studies, case reports and controlled trials. The keywords utilized in our search criteria include “Amyotrophic lateral sclerosis,” “assistive robotics,” “rehabilitation,” “exoskeleton,” “communication aid,” “adaptive device,” “quality of life.”

Of our initial 119 articles, 47 were excluded as they did not meet the scope of this project. Our main body of research [25–96] consisted of primary research such as pilot studies, case reports and controlled trials. Of those 72 articles, 52 publications [25–76] fully met our criteria involving device implementation in an ALS- specific setting, measuring subjective outcomes and quality of life improvements. The other 20 primary articles [77–96], either did not involve device implementation or utilized assistive robotics in a mixed population, not specifically addressing ALS.

### Robotics for Mobility Support

**Figure 1:**
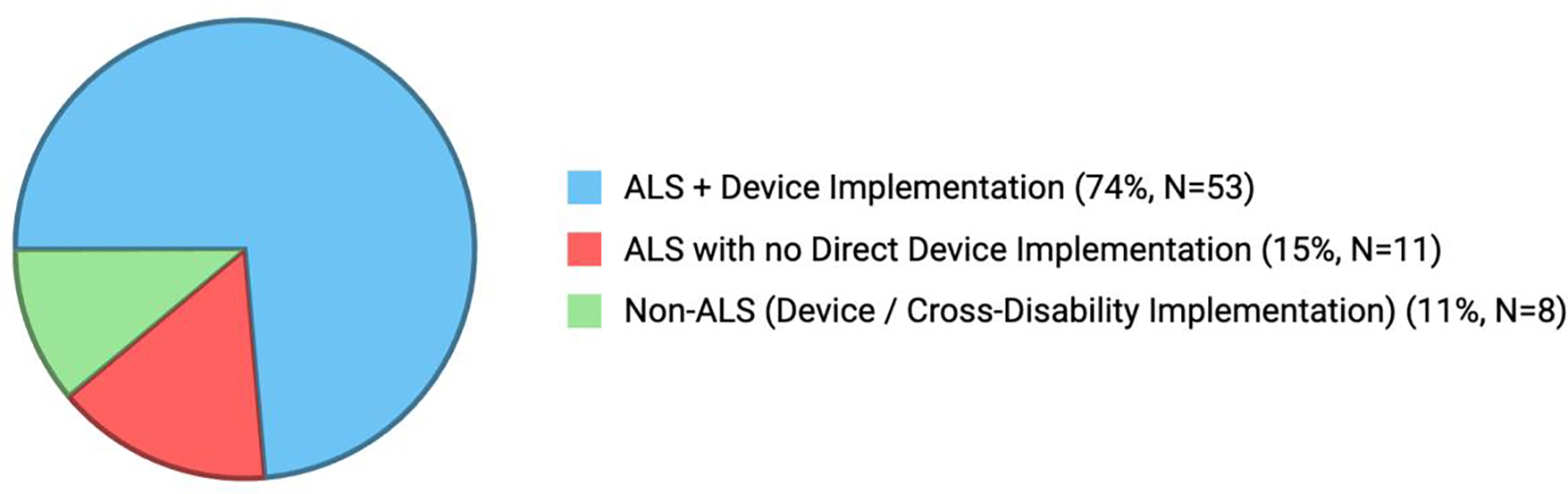
Current distribution of our primary body of research. The published articles and studies are categorized by their inclusion of assistive technology and focus on ALS. Sample size and percentage of total studies (N=72) in the individual category are shown in parentheses.

**Figure 2:**
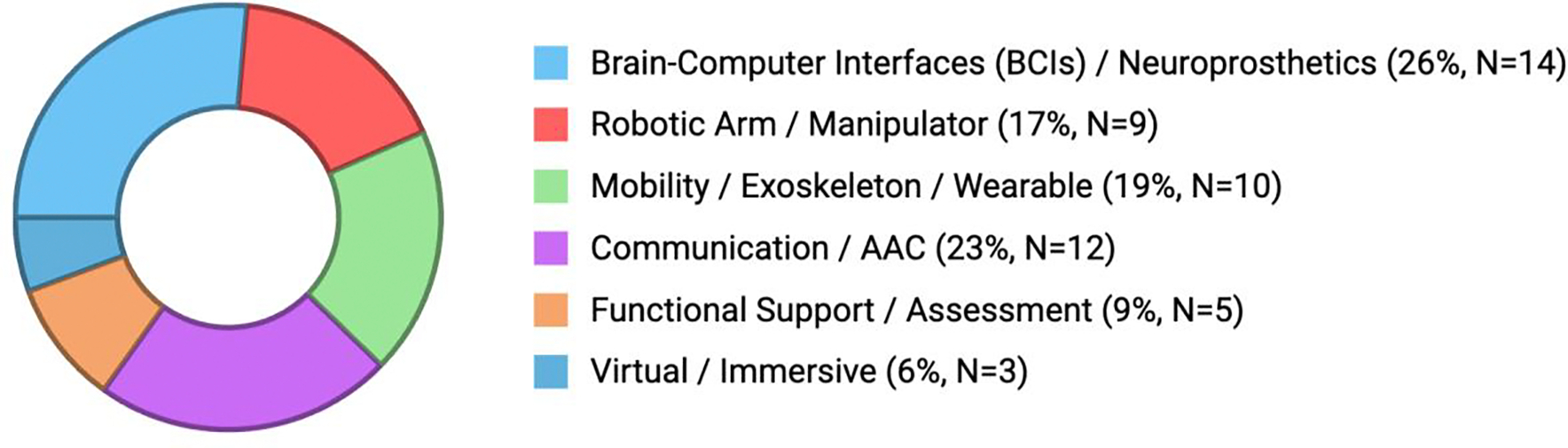
Distribution of our Primary ALS studies which utilized the implementation of assistive devices. The studies are categorized by technology domain. Sample size and percentage of total studies (N=53) in the individual category are shown in parentheses.

Motor decline in ALS is often experienced as a slow separation between intention and motion. Strength weakens, reflexes sharpen, and spasticity reshapes movement into something effortful and unpredictable. Posture begins to fail in ways that are subtle at first and then sudden all at once. Holding the head upright becomes tiring; standing or walking turns into a series of careful negotiations. Even shifting in a chair can require strategy. What was once automatic must now be managed moment by moment. It is within this erosion of stability and spontaneity that mobility-oriented robotics has begun to offer a different way forward. These technologies represent a substantial portion of contemporary ALS research. As shown in Figure 1, nearly half of all primary studies focus on restoring or supporting physical function, with mobility systems forming a major subdomain. Their growth over time is equally striking. Figure 2 reflects an accelerating trend toward wearable and soft-robotic designs; devices that do not aim to overpower the body but to move with it. This shift in research emphasis mirrors a philosophical one: a move away from rigid mechanical compensation and toward dynamic, adaptive support that respects how people live in their bodies.

Wearable and soft exoskeletons have reshaped expectations of what mobility assistance can feel like. Recent projects demonstrated that a fabric-based upper-limb exosuit could restore partial arm movement without restricting comfort or natural joint motion [25]. Zhou and colleagues expanded this concept with an inflatable shoulder robot that improved endurance and supported elevation tasks with minimal bulk [77]. Meanwhile, complementary work by Yamakawa et al. showed that a robotic glove improved finger dexterity and even modulated functional connectivity, suggesting neurophysiological benefits intertwined with mechanical support [34]. As these systems grow lighter and more compliant, they become easier to integrate into daily routines, an essential step for meaningful real-world adoption. Neck and shoulder robotics have followed a similar evolution. Demaree et al. [30] reported that redesigned neck exoskeleton structures help reduce fatigue and preserve alignment during functional tasks, while Zhang et al. [31] demonstrated that a powered cervico-thoracic orthosis can restore head control in individuals with ALS. This may seem like a small gain on paper, but in practice, head stability underlies communication, swallowing safety, and the ability to remain socially engaged. When patients can hold their gaze, they can participate.

Gait and trunk stabilization studies reinforce this momentum. Recent articles found Hybrid Assistive Limb (HAL) training improved balance and gait performance across neuromuscular conditions, including ALS [32]. Additionally, reductions were observed in compensatory movements and safer ambulation after robot-assisted gait interventions [29]. These improvements may be modest in clinical scale scores, but they carry immense significance in daily experience. A person who can rise from a chair more safely, hold their torso upright during conversation, or take several supported steps remains present in their world.

Across this entire domain, the trend is unmistakable. Mobility robotics are both expanding in volume and shifting toward softer, more human-aligned architectures. In doing so, these devices lay the groundwork for functional independence.

### Impact on Functional Capabilities in Daily Life

Function in rehabilitation is ultimately measured by what a person can do in the world. It extends beyond isolated movement, beyond range of motion or strength scores, into the everyday tasks that give shape to a life. In ALS, these Activities of Daily Living (ADLs)—feeding, reaching, turning a page, grasping a cup, adjusting clothing—are often among the first abilities to slip away as fine motor control weakens and coordinated task execution becomes difficult [12, 14]. The loss is not only practical. It is intimate. It is the quiet unthreading of routines that anchor independence. Assistive robotics designed for upper-limb and task-level support attempt to hold that thread a little longer, converting residual neuromuscular or cortical signals into meaningful action [20].

Across our dataset, upper-limb and task-oriented devices stand out as some of the most deeply explored technologies, which is not surprising. Preserving the ability to carry out daily tasks is often what matters most to patients and to the clinicians who walk with them through the course of ALS. Klebbe and colleagues [26] showed that robotic arm systems could help people complete essential ADLs, feeding themselves, moving objects, interacting with the space immediately around them, actions that shape the flow of an ordinary day. This was further echoed in a recent multicenter study, finding that participants valued more than the mechanical success of a task. What mattered was doing it on their own terms, in their own rhythm [27]. And when you look at longer-term observational work, this story only becomes clearer and more textured. This year alone, studies on the JACO robotic arm described a sense of restored agency—the opportunity to act without waiting, negotiating, or depending on another person for every small task [78–79]. Independence, even in fractional forms, became a source of confidence.

Intention-assisted and semi-autonomous systems further extend this capacity. Maier’s work with 3-D vision–guided manipulators revealed that these robots could interpret user intent with minimal cognitive or physical load [28]. Building upon that, Manero and colleagues [52] demonstrated that even faint surface electromyographic signals—sometimes barely detectable—could reliably drive motorized wheelchair control [52]. These hybrid systems preserve functionality in a disease that steadily takes it away, blurring the line between biological limitation and mechanical possibility.

Clinically, assistive robotics have shown a profound impact on patients’ functional capacity to perform ADLs. Portaro et al. [33] showed that a wearable upper-limb device improved the ability to perform reaching tasks in individuals with flail arm phenotype ALS. In addition to that, Morioka et al. [29] demonstrated that robot-assisted training not only supported gait but reduced the compensatory upper-limb strain that often interferes with daily activities. These experiences align with a broader trend across our figures: as shown in Figure 2, the field is steadily moving toward systems that support the practical, everyday dimensions of living.

**Figure 3:**
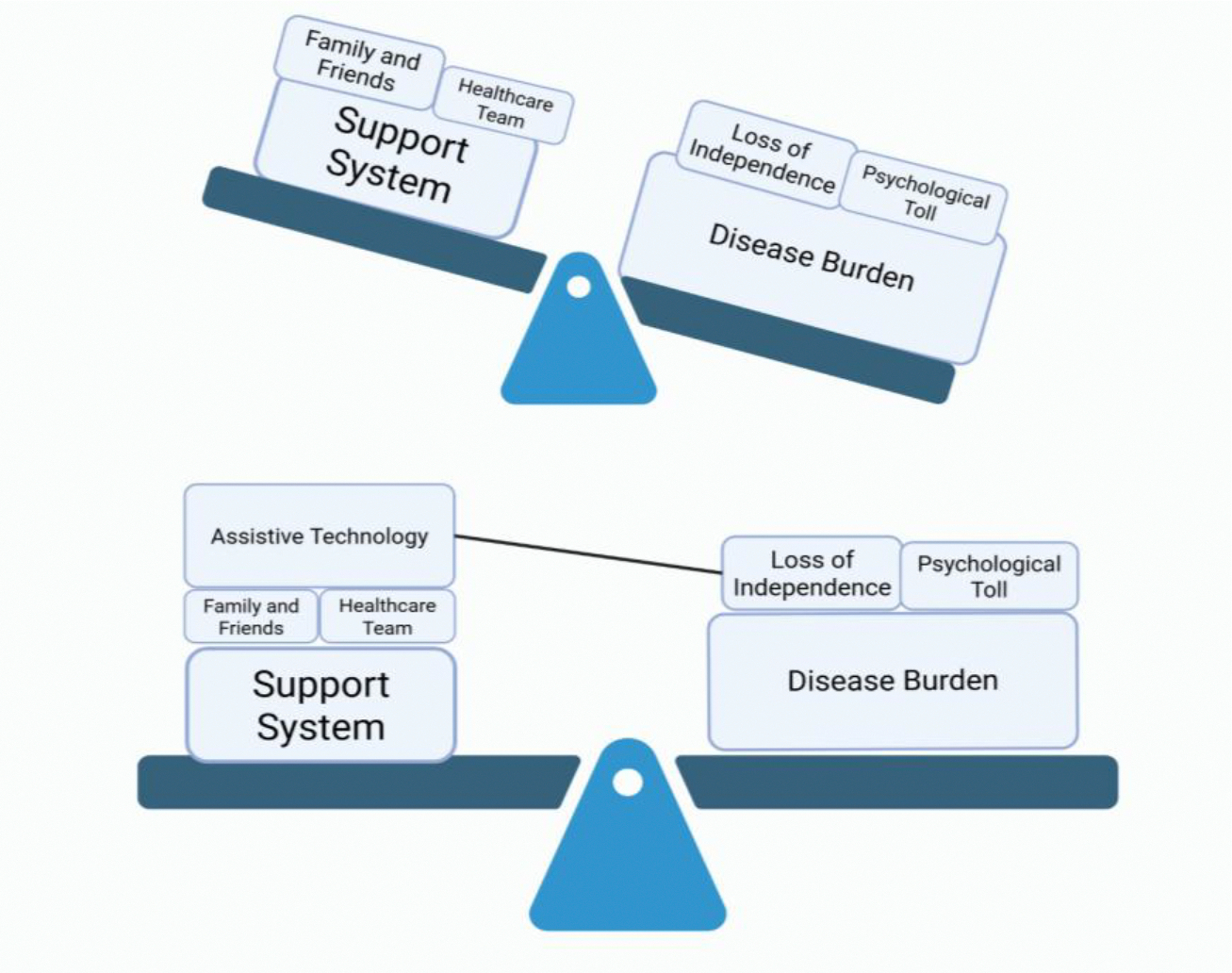
Visual Representation of the Impact Assistive technology has on improving QoL by alleviating burden on support system, and empowering patients with restored autonomy to some degree.

This shift is captured conceptually in Figure 3, visualizing how assistive technology alters the balance of care. On one side lies the growing weight borne by caregivers as ALS progresses—lifting, feeding, positioning, adjusting, anticipating. On the other side lies the patient’s diminishing autonomy. Introducing functional robotics does not flatten this seesaw entirely, but it changes its angle. By restoring even partial capability in ADLs, these devices lighten the caregiver’s load and raise the patient’s side of the balance. They create space for shared dignity: the caregiver is relieved of constant physical strain, and the patient regains the ability to participate in their own life.

From the perspective of rehabilitation medicine, this is the heart of functional support. ADLs are more than tasks; they are expressions of identity. They carry the rhythm of daily living and the texture of self-sufficiency. When assistive robotics protect a patient’s ability to act—to reach, to grasp, to feed, to manage their environment—they are not merely completing movements. They are preserving participation. And participation remains the essence of function, even as the disease progresses.

### Robotics for Communication and Cognitive Interfaces

When surveying the most important factors relating to quality of life in patients with ALS, psychological, spiritual and social aspects were cited as most important. While quality of life in patients with ALS was found to be independent of physical function [15]. One exception to physical functioning being independent of measures of physical function was speech impairment. Speech is an important part of maintaining relationships which are vital to the psychological, spiritual and social identity of patients. Speech impairments were found to have a significant impact on the perceived quality of life of patients. Quality of life was specifically found to be less in patients with mild speech impairment and total speech impairment as compared to patients with no speech impairment [13–14].

New developments in the assistive devices used for speech impairment and dysarthria introduce a new opportunity to care for patients with ALS in areas that will have a large impact on patient function and experience, and most importantly are an opportunity to improve quality of life in patients with ALS. Two promising and emerging technologies include eye tracking computer systems and brain computer interfaces. In comparison to traditional speech rehabilitation therapy and more rudimentary forms of augmentative communication such as alphabet boards, these devices allow for patients with lower levels of physical functioning to communicate independent of care takers. This is important as while quality of life is independent of physical function in patients, there is a decrease in quality of life in caretakers as patient physical function declines [14]. In one case study of a patient in the locked-in state due to ALS both eye tracking and an auditory brain computer interface were evaluated by the patient. The patient was able to use both forms of communication. The patient showed preference for his previous partner scanning approach to communication as compared to eye tracking software. However, they indicated that they would consider a brain computer interface if they were unable to use their current low-tech form of alternative communication [40]. In multiple studies of patients surveyed already using the eye tracking computer devices, and those with quadriplegia a high level of user satisfaction, increased psychological well-being and most importantly preservation of communication abilities were found [35, 37]. In a separate study eye tracking assistive devices were also shown to have a high improvement on the burden of caregivers [38]. These studies highlight the utility of eye tracking assistive communication devices, and the importance of creating strategies for device adoption. Evaluation of eye tracking assistive communication devices found that most regular users were younger and disease onset, and low utilization was related to eye-gaze tiredness [39].

Currently eye tracking devices are more widely used than the more recent brain computer interfaces, in addition early brain computer interfaces were found to be more fatiguing [40]. Promisingly brain computer interface technology was found to have greater accuracy in spelling tasks as compared to commercially available eye tracking [41]. In conjunction with the potential of brain computer interfaces in patients with more severe motor dysfunction, the potential to rehabilitate previously unattainable function in ALS patients such as with brain painting have sparked greater interest in development [64]. Specific to communication brain neuroprosthesis have been shown to be able to replicate intonation, and paralinguistic features to patient speech [65]. The accuracies of these technologies have been shown to be incredibly accurate. In a 45-year-old man with ALS who had a BCI in 1 day there was a 99.6% accuracy with 50-word vocab, 90.2% accuracy in 125,000 vocab and was able to sustain a 97.5% accuracy over 8.4 months with 32 words per minute over 248 combined hours. Brain computer interfaces were found to be heavily used compared to other forms of communication when available [47]. Brain computer interfaces were also found to be progressively more important as disease progresses. In one patient who used the device for seven years. As the patient’s motor function declined and was progressively unable to use eye tracking technology, patients’ use of the device increased as a substitute [46]. In another case a patient reported high satisfaction with the brain computer interface in independent home use. It also showed longitudinal potential in brain painting and was found to be less straining than an eye tracker in this application, which allowed one woman to even display and sell paintings made with the software [49]. Further development should be done into developing brain computer interfaces for ALS patients. When surveyed, patients with ALS cited accuracy, and the ability to use technology without surgery with anesthesia were major things they would want in future brain computer interface technology [66]. Furthermore, future research in larger populations as well as in feasibility of cost and training should be done.

### Quality of Life and Human-Machine Integration

Integrating assistive robotics into the care of people living with ALS is more than a technical achievement; it’s an act of compassion and imagination. The true challenge isn’t found in circuits or algorithms, but in the deeper question of care itself: how do we help someone continue to move, to speak, to remain part of the world, even as their body grows quiet?

Our current care system has begun to focus beyond replacing lost function; they extend the dialogue between human and machine into the fabric of daily life. Maier et al. [28] and Manero et al. [52] demonstrated that electromyographic and semi-autonomous control systems could sustain motor agency when voluntary strength waned. Meanwhile additional projects revealed that cortical interfaces could bypass the body entirely [42, 45–47]. Each of these advances speaks to a deeper form of integration, not merely using devices but incorporating them into the lived identity of care.

At the heart of this evolution is quality of life. Across our dataset, nearly three-quarters of primary studies measured psychosocial or independence outcomes alongside technical metrics. Participants in Spittel et al. [27] and Gitlow et al. [28] valued reliability, comfort, and familiarity more than speed or precision. For many, their robotic arm or exoskeleton became an extension of self; a means of reclaiming routines that define humanity more than motion itself. Similar findings revealed that users often described these technologies not as tools, but as companions [35, 37, 59, 73]. They reintroduced presence into everyday rituals; holding utensils, maintaining eye contact, or sitting upright during conversation; transforming assistance into participation.

At the clinical level, bionic integration succeeds when it is designed as a continuum of care rather than a one-time intervention. Tirschmann et al. [54] and Lucassen et al. [55] emphasize multidisciplinary collaboration. Engineers refining devices alongside neurologists, therapists, and caregivers who understand the nuances of fatigue, posture, and adaptation would be the hallmark of this intersection. At ALS specialty clinics, robotics now intersects with respiratory therapy, occupational training, and palliative planning. The technology’s success thus depends as much on empathy as on circuitry and its ability to fit seamlessly into a patient’s daily rhythm without overshadowing their humanity [89, 96].

**Figure 4:**
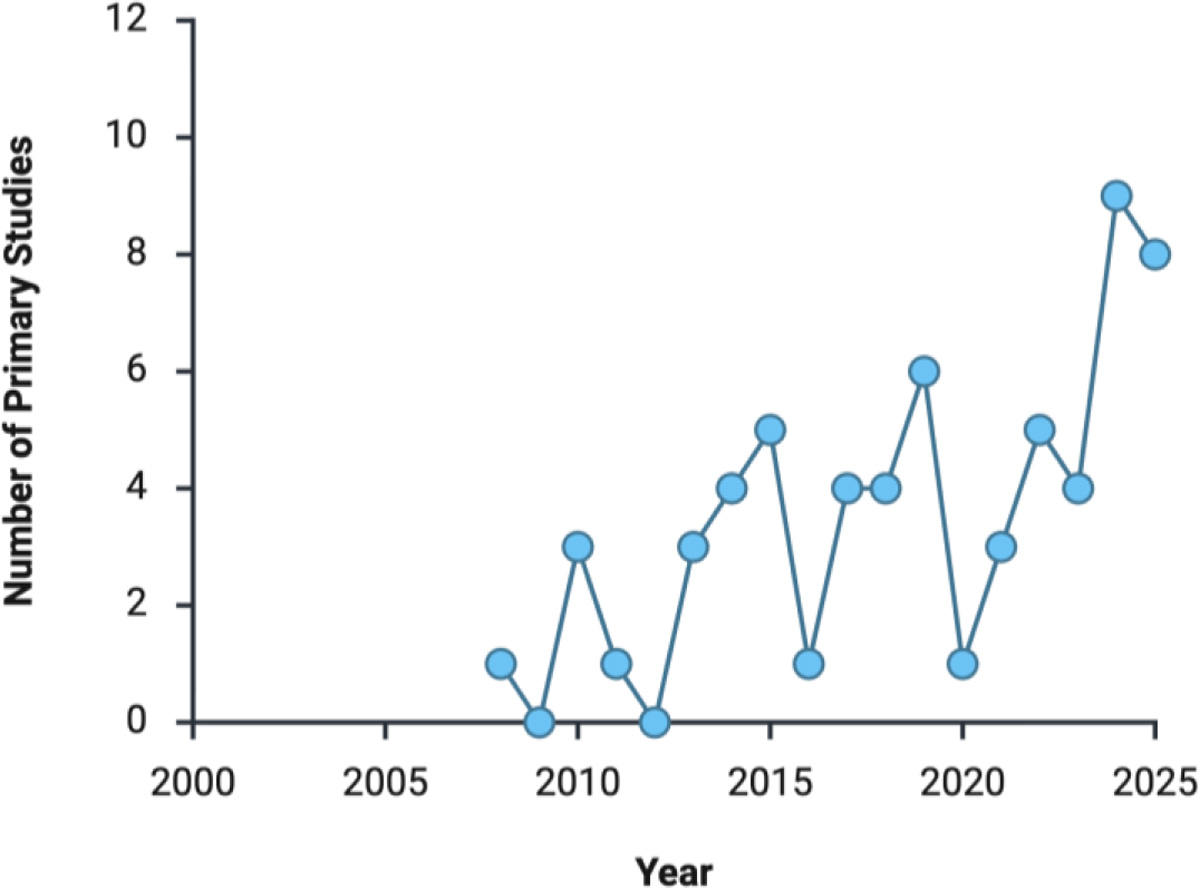
Timeline of the current scope of primary research involving ALS and the implementation of assistive technology (n=53). The number of primary studies demonstrates the emergent nature of this field, and the need for more robust studies conducted in this subset of the field of Assistive technology.

The trajectory of the literature mirrors this philosophical shift. From 2008 to 2018, only a handful of studies referenced emotional or social outcomes. By contrast, since 2020, the proportion of papers addressing quality of life, user experience, or caregiver well-being has more than doubled, reflecting a field that now measures meaning alongside mechanics. Figure 4 illustrates this temporal trend: a steady rise in QoL-centered publications, culminating in 2024–2025, when such metrics became standard endpoints in nearly one-third of ALS robotics research. This trend parallels the technological maturation seen in Figures 1 and 2, highlighting the shift from feasibility into integration.

Human–machine integration therefore represents a new kind of partnership; one that is not purely mechanical but existential. In the hands of clinicians, engineers, and patients, robotics has become a language of continuity. Each mechanical gesture becomes an assertion of personhood. Through this lens, technology in ALS is not a monument to loss but a testament to adaptation. When the nervous system grows silent, the human spirit still speaks, carried forward by the quiet precision of machines built to understand it.

### Barriers and Ethical Considerations

Despite the incredible potential, the field of assistive robotics has for the management of ALS, barriers persist. There remains an intricate web of economic, ethical and scientific barriers. Most notably, economic and accessibility constraints determine the beneficiaries from this progress.

For most patients, the financial cost of these innovations is daunting. Insurance coverage offers little relief, shaped by policies that favor what preserves function over what preserves joy. Devices that help a patient breathe may be funded; those that help them eat or write or gesture may not.

As Connolly et al. [56] and Lucassen et al. [55] observed, patients rarely abandon assistive technologies because they lose hope. More often, the cause is marked by cost, logistics, or the lack of consistent technical support. Each discontinued device represents not failure, but fatigue: a system unable to sustain the very independence it promises. Globally, the divide widens still further. The wealthy nations who can develop prototypes prosper while other parts of the world lack basic adaptive communications tools.

This imbalance is not only economic; it is moral. The capacity to move, to communicate, to act with agency should not depend on geography or income. True innovation must therefore reach beyond design to justice; establishing frameworks that recognize mobility and communication not as privileges, but as essential expressions of human dignity. Vansteensel et al. [46] and Card et al. [47] highlight that long-term cortical interface use raises unresolved questions about data ownership, cognitive privacy, and control. As ALS progresses, these tensions deepen. End-of-life care complicates the ethics of continuation versus comfort: at what point does prolonging technological engagement begin to overshadow dignity? These are not abstract questions but practical considerations that shape how care teams and families navigate the moral boundaries of innovation.

Lastly, there remains a distinct gap in the current literature for this field. Despite recent rapid growth in publication, many of these studies are small, non-randomized and limited in longitudinal scope. Our analysis of recent primary studies showed that almost 40% consisted of single-case investigations or pilot studies. Standardized data and variability in the metrics made cross-study comparisons difficult. Burke et al. [8] and Proietti et al. [25] emphasize that the absence of unified evaluation frameworks constrains evidence synthesis and hinders translation into clinical guidelines. Amidst the field of assistive robotics, ALS remains underrepresented, a bulk of the current robotics literature focused on stroke and spinal cord injury patients.

**Figure 5:**
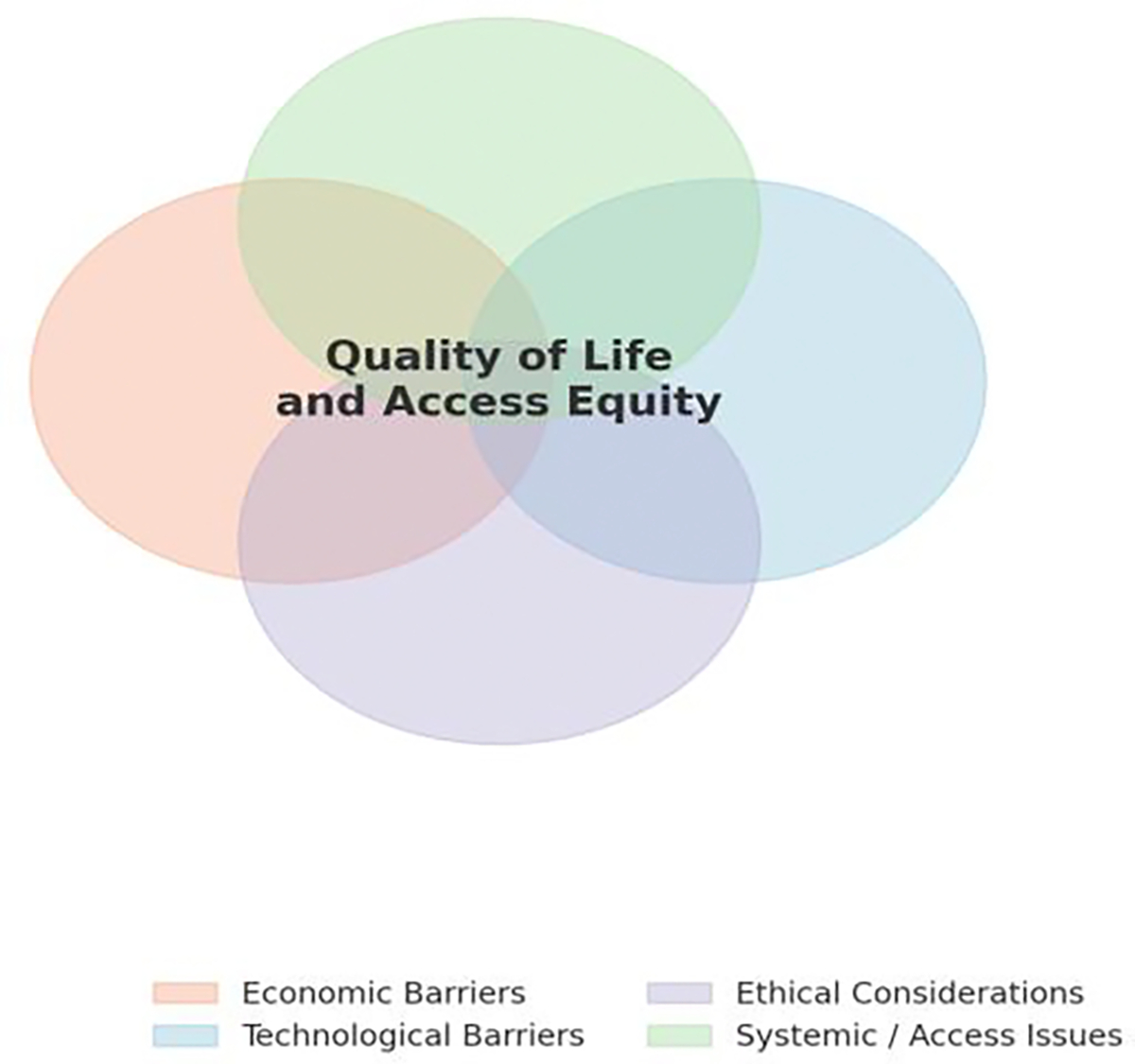
Interacting Barriers and Ethical Dimensions in ALS Robotics Integration. This conceptual diagram illustrates how economic, technological, ethical, and systemic factors overlap to shape patient outcomes in assistive robotics for ALS.

**Figure 6:**
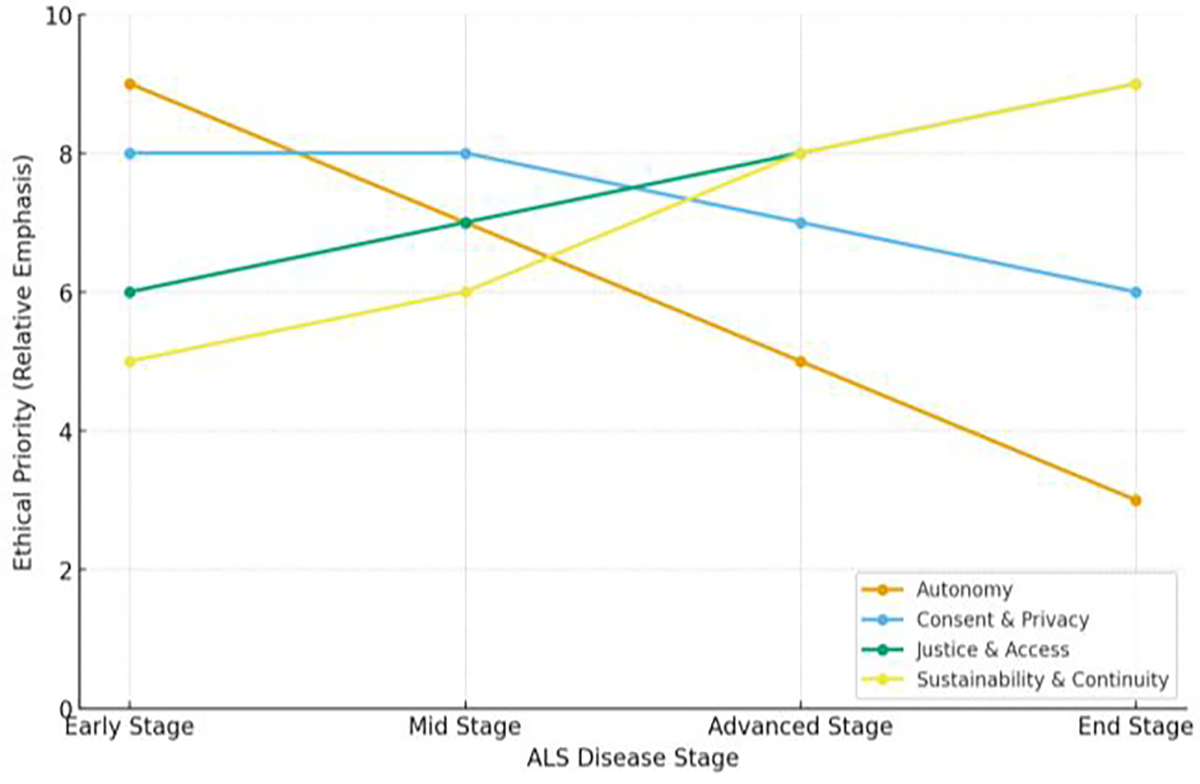
Conceptual framework illustrating the shifting emphasis of ethical priorities across ALS disease progression. Relative values are derived from thematic synthesis of published literature (2008–2025) and reflect interpretive trends rather than empirical measurement.

When examined together, these barriers reveal the multidimensional nature of the challenge. Figure 5 shows how economic, ethical, and technological factors converge around the patient’s quality of life. Effectively, this demonstrating that limitations in one domain amplify inequities in the others. Furthermore, Figure 6 expands upon this perspective across the disease timeline, illustrating how ethical priorities shift as ALS progresses. The barriers to implementation are not isolated. They interact, evolve, and ultimately determine the degree to which robotics can achieve meaningful human integration.

### Future Directions and Policy Implications

The next stage of assistive robotics in Amyotrophic Lateral Sclerosis will depend not only on innovation, but on integration, on how well technology can be woven into care, policy, and the daily rhythm of human life. Engineering has already transformed what once felt impossible. Robotic arms now restore the ability to grasp. Exosuits stabilize the weight of a collapsing shoulder. Brain–computer interfaces give voice to those who can no longer speak. [25, 46–47] Yet progress cannot end with invention. The real measure of this field lies in how these devices are lived with — how naturally they join the continuum of multidisciplinary care, how deeply they preserve dignity, and how consistently they reach those who need them most.

Integration begins with collaboration. Engineers and clinicians already share the same goal, though they often speak different languages. Studies by Spittel et al. [27] and Tirschmann et al. [54] remind us that outcomes improve when design is guided by rehabilitation insight and lived experience rather than technological ambition alone. Robotics should never stand apart from therapy, but move with it — evolving alongside the person, not in parallel to them. In osteopathic and rehabilitation medicine, this principle feels intuitive: structure and function are one. Even when movement is sustained by machinery, it remains an expression of the human whole — body, mind, and intention intertwined.

There is also a tremendous horizon for research. Despite recent growth, much of the literature still focuses on short-term feasibility trials [8, 28]. The next decade demands longitudinal studies. Real-world implementation trials are needed too; research that measures how devices perform not in laboratories, but in living rooms. Comparative analyses across conditions like stroke or muscular dystrophy could also reveal shared mechanisms of neuroplasticity and recovery. As Tzeplaeff et al. [2] and Pugliese et al. [10] argue, the field now needs more than imagination; it needs evidence, robust, reproducible, and ready to inform policy and reimbursement.

Yet even as the field matures, progress must stay rooted in ethics. The convergence of robotics, artificial intelligence, and neural data has outpaced the systems built to regulate it. Patient-centered design should be the rule, not the exception. Co-creation with patients and caregivers, as Lucassen et al. [55] and Tirschmann et al. [54] emphasize, keeps technology grounded in human experience, enhancing agency rather than deepening dependency. Equity in access must move from aspiration to obligation. Sustainable innovation will depend on open-source designs, modular manufacturing, and international partnerships that reduce cost while increasing adaptability. These models make robotics not only more affordable, but more human; designed for inclusion rather than exclusivity.

Ethical clarity must guide this evolution. Vansteensel et al. [46] and Card et al. [47] remind us that as technology listens to the mind, it must also learn restraint. Questions of privacy, ownership, and end-of-life care belong not to manufacturers, but to communities of care, to clinicians, patients, and families who must decide together when technology serves life and when it should yield to comfort.

In the end, the path forward is as much moral as it is mechanical. Robotics may extend the body’s reach, but it must also extend the reach of compassion. The next decade will test whether innovation can coexist with empathy, whether precision can be joined to purpose. True progress in ALS will not be measured by movement alone. It will be measured by meaning, by how gently technology helps patients remain themselves, and how deeply it reaffirms that the work of medicine, at its best, is to restore connection where decline once seemed inevitable.

## Conclusion

Assistive robotics shows immense potential in impacting the field of ALS management. Across decades of research and human effort, these technologies have offered something the disease was never meant to allow the chance to retain movement, voice, and agency even as the body changes. From robotic arms that restore independence in feeding to brain–computer interfaces that translate thought into communication, each innovation represents more than mechanical progress. These new innovative technologies reveal a pathway to preserving autonomy, dignity, and meaningful participation in life.

Yet the promise of robotics is not measured by engineering alone. It is measured by how well these tools align with the deeper work of care, to preserve the self within the illness. The studies reviewed here remind us that progress in ALS is never simply technical; it is moral. Devices can sustain movement, but they must also sustain belonging. Systems can improve survival, but they must also protect quality of life. Between the lines of every trial and case report lies the same question: can technology serve humanity without overshadowing it?

Hope in this field is not naïve; it is necessary. The science will continue to advance, more precise control, lighter materials, adaptive interfaces that learn from the user, but its success will depend on whether it grows with empathy. To move forward, robotics should be visualized as an extension of care by clinicians. Engineers must design with patients, not just for them. Policymakers must craft frameworks that recognize movement and communication as rights, not privileges. And patients themselves, through their courage and insight, must remain central to this dialogue, shaping the innovations that shape their lives.

The challenge ahead is profound, but within it lives a quiet hope. ALS will always test the limits of the body, yet how we respond — as clinicians, engineers, and human beings — remains a choice. Through collaboration and adaptability, technology can become more than a tool; it can become a bridge. It can carry a person through the spaces where muscle fails but spirit endures. With every act of design grounded in respect, the experience of decline begins to change. It becomes less about what is lost and more about what can still be held — continuity, connection, and care. In the end, the future of assistive robotics is not about outpacing mortality. It is about preserving what gives life its shape and texture: meaning, presence, and our shared humanity.

## Key points:

- ALS gradually disrupts the connection between intention and movement, and understanding this clinical trajectory clarifies why assistive robotics are becoming increasingly essential across stages of disease.
- Mobility-focused robotics help preserve posture, stability, and head control, allowing patients to remain safely engaged in their environments even as motor decline progresses.
- Functional support technologies restore everyday Activities of Daily Living, enabling patients to feed themselves, reach for objects, and participate in meaningful routines that anchor independence.
- Communication tools, including advanced eye-tracking systems and brain–computer interfaces, protect the patient’s ability to express thought and preference long after speech is lost.
- Successful human–machine integration depends on intuitive, adaptive systems that respect the patient’s lived experience and align with multidisciplinary clinical care.
- Assistive robotics can improve quality of life by reducing caregiver strain and restoring a degree of autonomy, shifting the balance of support toward shared relief and empowerment.
- Barriers such as cost, inequitable access, insurance limitations, and ethical concerns about privacy and autonomy continue to restrict widespread use despite strong evidence of benefit.
- Emerging research trends show a clear movement toward soft, wearable, and user-centered designs, signaling a shift from laboratory feasibility to real-world usability and comfort.
- Future progress requires longitudinal studies, home-based implementation trials, and comparative research across neuromuscular diseases to strengthen evidence and guide clinical adoption.
- Policy reform is essential to ensure that assistive robotics are treated as fundamental disability supports; with equitable access, sustainable funding, and ethical oversight built into systems of care.
